# Stress, Mental Health, and Risk-Taking: Associations among a Sample of Agricultural Adolescents

**DOI:** 10.3390/ijerph21070830

**Published:** 2024-06-26

**Authors:** Josie M. Rudolphi, Richard L. Berg

**Affiliations:** 1 Department of Agricultural and Biological Engineering, University of Illinois Urbana, Champaign, IL 60801, USA; 2 Office of Research Computing and Analytics, Marshfield Clinic Research Institute, Marshfield, WI 54449, USA

**Keywords:** agriculture, adolescent, stress, depression, risk-taking, substance use, violence

## Abstract

Farm stress and mental health research has largely focused on adult producers, even though youths live and work on farms. The purpose of this study is to describe the stress and mental health experience of agricultural youths and describe participation in risk-taking behaviors. Farm families in the U.S. were invited to participate in an online survey that inquired about farm economics, stress (ASQ-S), mental health (PHQ-A and SCARED), parenting, social support, and risk-taking behaviors during the summers in 2021 and 2022. This analysis is limited to adolescent respondents (*N* = 298). Just over 70% of the farm adolescent sample met the criteria for at least mild symptoms of depression (PHQ-A score ≥ 5). Among anxiety disorders, 62.8% of the farm adolescent sample met the criteria for probable panic disorder, and 70.5% met the criteria for probable separation anxiety disorder. Adolescents reported the most stress around future uncertainty and school performance. The stress scores were significantly associated with more symptoms of depression. Over a quarter (27.7%) of the participants reported carrying a weapon at least 1 day in the past month, and 22.5% reported carrying a weapon on school property at least 1 day in the past month. Violence-related risk-taking behaviors were also associated with increased symptoms of depression. The results underscore the need for community- and school-based stress and mental health interventions in rural and agricultural communities.

## 1. Introduction

Agricultural producers experience worse mental health than the general population, characterized by a higher prevalence of anxiety and depression than the general population and increased suicide risk [1,2,3,4,5]. Research has characterized sources of stress among U.S. farmers, ranchers, and farmworkers; examined associations with mental health conditions; and described substance use among adult owner/operators and farmworkers [1,2,3,6,7,8,9,10,11,12,13,14]. However, the research and subsequent resource and service development and delivery have largely focused on adult agricultural producers and workers. Farm adolescents may represent a unique population with respect to their risk of poor mental health. 

There are an estimated 2 million youths living and working on farms in the United States. Farm youths notoriously participate in agricultural work at a young age and experience high rates of work-related injury, like their adult counterparts [15,16]. These youths may experience unique types of stress that contribute to poor mental health. Among adult farmers, personal finances, economic conditions, adverse environmental conditions, and interpersonal relationships are routinely mentioned as the leading sources of stress and have been associated with symptoms of anxiety and depression [8,9,10,17]; however, the same research has not been conducted among youths. The farm is a place of work but also a place of residence. Given the presence of youths in the farm environment, as workers or residents, their exposure to the stressful realities of agricultural production may impact their mental health. 

Regardless of farm status, adolescence is a formative time, marked by physical, mental, emotional, and social changes [18]. In the U.S., adolescent mental health is a public health priority. Common mental health disorders among youths in the U.S. include depression, anxiety, and behavioral disorders [19]. In a given year, it is estimated that approximately 15.1% of adolescents aged 12–17 years old in the U.S. experience a major depressive episode, defined as a “period of at least two weeks when a person experienced a depressed mood or loss of interest or pleasure in daily activities, and had a majority of specified symptoms” [20], and 5.8% experience self-reported symptoms of at least moderate depression based on the Patient Health Questionnaire-9 (PHQ-9) screening instrument [21]. Similarly, among high school students in the U.S., 36.7% reported persistently feeling sad or hopeless in the past year [21]. 

While mental health disorders affect children and adolescents across a range of demographic and socioeconomic characteristics, the prevalence varies by certain characteristics. Girls have consistently shown higher estimated prevalences of depression, suicidal ideation, and attempted suicide when compared to boys [22] but a similar prevalence of anxiety [21]. When compared to urban adolescents, rural adolescents were slightly more likely to have had depression diagnoses, as reported by a parent, or current depression, also reported by a parent, but there were no notable differences in self-reported major depressive episodes ever or in the past year [21]. However, there is limited research specifically describing the mental health experience of agricultural youths. 

Conditions and exposures during adolescence, such as poverty, violence, and stress, can make young adults vulnerable to mental health problems. Stressful experiences during adolescence, such as exposure to adversity, pressure to conform, quality of their home life and relationships with peers, harsh parenting, and socioeconomic problems, are recognized risks to mental health [23,24,25]. Negative economic conditions increase stress among parents and increase intrafamily conflicts [26]. Financial-related situations such as a reduction in income and wealth can affect children’s mental health [27]. Agriculture is financially turbulent, characterized by unpredictable and unstable markets, fluctuating interest rates, and high debt loads. People in agricultural production often experience inconsistent incomes and irregular paydays. Rural adolescents may face unique experiences that exacerbate their mental health, including limited social networks, geographic isolation, and limited access to mental health resources [28]. Given their exposure and participation in agriculture and the fact that they often reside in rural communities that may lack mental health resources, farm youths may be at high risk of adverse mental health conditions. 

Adolescents’ health and well-being are further threatened by their inclination to engage in risky behaviors [18]. Importantly, risk-taking behaviors among adolescents are associated with negative mental health outcomes, including symptoms of anxiety and depression [29,30]. Risk-taking behaviors characteristically involve participation in behaviors that increase the likelihood for injury, comorbidity, or mortality [31]. Behaviors such as violence, substance misuse, and failure to take safety precautions, while considered part of the adolescent experience in many populations, can impact an adolescent’s mental well-being. Rural youths may be more inclined to participate in risk-taking behaviors when compared to urban youths. Rural adolescents have higher rates of substance use, such as alcohol and inhalants, and are more likely to engage in dangerous behavior, such as binge drinking and driving while under the influence of a substance [32]. 

An analysis comparing rural farm to rural, non-farm youths found farm males reported more engagement in risk-taking behaviors, including behaviors such as smoking, consuming alcohol, non-bicycle helmet use, and energy drink consumption. Similarly, farm females were more likely to engage in these risk-taking behaviors than rural, non-farm females [33], though the differences were generally not as strong. Importantly, risk-taking was strongly and consistently associated with negative indicators of mental health, including lower life satisfaction and psychosomatic symptoms [33]. 

Much of the existing research on the mental health of farm adolescents is from outside of the United States. The objectives of this analysis are to describe sources of stress among a U.S. sample of agricultural adolescents and explore the association between stress, mental health, and risk-taking behaviors by sex. 

## 2. Materials and Methods

This cross-sectional study was conducted among agricultural producers and their adolescent children during the summers of 2021 and 2022. All research protocols were reviewed and approved by the Institutional Review Board at the University of Illinois Urbana-Champaign on 21 April 2021 under protocol #21759. 

### 2.1. Methods 

The target population was farm families with at least one adult and one adolescent between the ages of 13 and 18 in the household. Initially, recruitment was focused on a five-state region (Illinois, Iowa, Wisconsin, Minnesota, and Indiana). These five states are among the most agriculturally productive, have a high proportion of primary producers that report farming as their full-time operation, and have a high proportion of primary operators that reside on the farm they operate [34,35]. The target population was later expanded to include farm and ranch families from the U.S. regardless of their state of residence. 

Two primary strategies were employed to recruit farm families into the study: mail and online. Annually, a list of addresses of 1000 agricultural producers in five Midwestern states with at least one adolescent in the household was purchased from US Farm Data (previously Farm Market ID). US Farm Data maintains a list of farm owners and operators based on the same sources as the U.S. Department of Agriculture’s (USDA’s) databases and estimates 95% coverage of farm owners and operators. US Farm Data overlayed the USDA’s agricultural census variables with consumer variables to identify primary producers who work on a farm full-time, live on the same farm they operate, and have at least one adolescent (aged 13–17) in their household [36]. Recruitment and survey materials were mailed to the 1000 agricultural producers using a modified Dillman approach to encourage a survey response [37]. Dillman encourages unique, repeated contacts with potential participants [37]. An introductory postcard to pique interest was sent to each address. Three weeks later, a packet of information followed that included the objectives of the study, IRB information, directions for participating, and a QR code to the online surveys. Three weeks later, a reminder postcard was sent that included the QR code to the online surveys. 

Recruitment also occurred online. The project team partnered with Extension, commodity groups, and farm organizations to disseminate information about the study via email, newsletters, and social media. Emails, newsletters, and social media posts directed individuals to an online study blog that described the objectives of the study, provided IRB information, directions for participating, and a link to the online surveys. Duplicate online surveys, with unique web addresses, were created to distinguish participants by recruitment method. 

The project team worked with the Research Electronic Data Capture (REDCap) administrators at the University of Illinois Urbana-Champaign to develop a series of consent forms and assent forms in addition to the online surveys to ensure the protection of the participants. REDCap is a secure, web-based application designed to support data capture for research studies, providing (1) an intuitive interface for validated data entry, (2) audit trails for tracking data manipulation and export procedures, (3) automated export procedures for seamless data downloads to common statistical packages, and (4) procedures for importing data from external sources [38]. 

A QR code and link on the study blog, provided on recruitment materials (letters, emails, and social media posts), led to an adult/parent consent form. The consent form collected name, email address, and signature of an adult/parent (P1). The form also consented an adolescent in the household to participate and collected the name and email address of the consented adolescent. Finally, the form inquired about a second adult in the household and asked for the name and email address of the second adult (P2), if applicable. After consenting themselves, consenting the adolescent in the house, and providing the contact information for a second adult (if applicable and optional), the adult proceeded to the P1 online survey. 

REDCap auto-emailed the adolescent a link to the assent form, which had to be completed by the adolescent prior to unlocking the adolescent survey. Similarly, REDCap auto-emailed the second adult in the household a link to their consent form and, ultimately, their online survey, the P2 survey. REDCap linked the family consent forms, assent forms, and surveys. All survey participants that completed the survey, met the inclusion criteria, and were deemed a legitimate response (non-bot respondent) were emailed an electronic Amazon gift card valued at 10 USD. 

Data collection occurred during the summer months in 2021 and 2022. This time period was selected because adolescents are on summer break in the U.S. and may be more involved in agricultural work. 

### 2.2. Materials

The Farm Stress and Mental Health Adolescent Survey was completed by farm adolescents and included the following sections and questions and/or instruments. 

#### 2.2.1. Screening Questions

Screening questions asked at the beginning of the survey determined the eligibility of the adolescents. To proceed with the survey, adolescents must have indicated they reside on a farm, ranch, or agricultural operation for at least 50% of the time and be between the ages of 13 and 17. 

#### 2.2.2. Demographic and Farm Characteristics

Adolescents responded to questions about age, school grade, race, state of residency, farm work status, hours per week involved in farm work during the summer and school year, and farm injury experience.

#### 2.2.3. Mental Health

Adolescents responded to the Patient Health Questionnaire-A (PHQ-A) to self-report symptoms of depression. The PHQ-A has demonstrated satisfactory sensitivity, specificity, diagnostic agreement, and overall diagnostic accuracy compared to a clinical interview [39]. Participants responded to nine statements that inquired about the frequency of depressive symptoms in the past two weeks (i.e., poor appetite or overeating and feeling tired or having little energy) on a Likert scale. The Likert scale response options were assigned point values ranging from 0 (not all) to 3 (nearly every day). PHQ-A scores were calculated by summing the values corresponding to each response. The possible score range was 0–27, with the following cutoff points to indicate severity: 0–4 was considered the normal range or full remission; 5–9 was considered minimal depressive symptoms; 10–14 was considered major depression, mild severity; 15–19 was considered major depression, moderate severity; and 20 or higher was considered major depression, severe severity [39]. 

Farm adolescents responded to the Screen for Anxiety Related Disorders (SCARED) to indicate symptoms of anxiety disorders. The SCARED consists of 41 items related to the 5 factors that parallel the DSM-IV classification of anxiety disorders: general anxiety disorder (9 items), separation anxiety disorder (5 items), panic disorder (7 items), social anxiety (8 items), and school phobia (3 items) [40]. Adolescents indicated how true each item was in the past three months on a Likert scale. Each Likert scale response was assigned a point value: 0 (not true or hardly true), 1 (somewhat true of sometimes true), and 2 (very true or often true). Scores for each anxiety-related disorder were calculated by summing the responses to the items within each factor. Cut-off scores indicating probable or likely anxiety varied by type of anxiety related disorder. Across all 41 items, a total score of ≥25 might indicate the presence of an anxiety disorder [40]. A score of 7 or greater on the items related to panic disorder might indicate a Panic Disorder of significant somatic symptoms. A score of 9 or greater on the general anxiety items might indicate Generalized Anxiety Disorder. A score of 5 or greater on items related to separation might indicate Separation Anxiety Disorder. A score of 8 or greater on the 8 items related to social situations might indicate Social Anxiety Disorder. A score of 3 or greater on the school phobia items might indicate significant school avoidance [40]. 

#### 2.2.4. Stress

The Adolescent Stress Questionnaire—Short (ASQ-S) evaluated sources of stress among adolescents. This is a 27-item inventory reflecting five dimensions of stress: home life (7 items), school attendance and teacher interactions (11 items), peer pressure (4 items), future uncertainty (3 items), and financial pressure (2 items). Adolescents indicated how much stress each item has caused in the past 12 months on a Likert scale (0 = not at all, 4 = very stressful). The dimension scores were calculated by summing the affirmed response to each item within each dimension and dividing the sum by the number of items within each dimension [41]. The permissible range of scores for each dimension was 0–4, with higher scores indicating more stress. The ASQ has been shown to be valid for measuring stress in research and clinical contexts [41,42].

#### 2.2.5. Risk-Taking Behaviors

Adolescents self-reported risk-taking activities related to substance use, sexual activity, and intentional/unintentional injury. Adolescents self-reported how often they participated in the following activities in the past 30 days and the past 12 months: number of times rode in a car or other vehicle driven by someone who had been drinking alcohol, seatbelt use when riding in a car driven by someone else, how many days they text or e-mail while driving a car or other vehicle, days they carried a weapon, days they carried a weapon on school property, days they carried a gun, days skipping school, and number of times in a physical fight [43]. Adolescents indicated whether they ever tried a substance (tobacco, marijuana, and alcohol); how old they were when they first tried the substance; how many days they used the substance in the past 30 days; and frequency of binge drinking in the past 30 days (if applicable). The following substances were inquired about: cigarettes; electronic vapor products; chewing tobacco; alcohol (included beer, wine, wine coolers, and liquors); marijuana; synthetic marijuana; and prescription medication for non-medication purposes [43]. 

### 2.3. Data Analysis 

In the final analysis cohort of 298 farm adolescents, relatively few response items were missing. For example, individual items in the PHQ-A were missed for, at most, 3/298 (1%) adolescents, while 4 of the 9 individual items showed no missing responses. To allow for some missed items, summary scores for each instrument were calculated whenever over ½ of the items were completed, and the mean of the completed items was used to scale the summary score to the same range as possible with no missing items. For the PHQ-A, all subjects completed at least 8 out of 9 items, and so all were included in the analyses.

Descriptive summaries were created to characterize the analysis cohort using standard descriptive statistics. Pearson’s chi-square was used to test for associations in the cross-tabulations, while the nonparametric Kruskal–Wallis test was used for group comparisons of numeric variables (e.g., the total over items in a scale). The results in this report were deemed statistically significant at the 5% level (*p* < 0.05) without adjustment for multiple comparisons.

Online recruitment resulted in a large number of suspected illegitimate responses, possibly robot-generated. A strict protocol to identify, flag, and remove suspect responses was implemented. A full description of this protocol has been described by the authors [44]. The total number of survey respondents during the two periods of data collection was 6157. However, only 1814 respondent families completed the surveys and provided the necessary consent documentation. Another 1516 were excluded because they were deemed illegitimate or bot-generated. 

## 3. Results

Of the 298 respondents, two-thirds were boys, and the mean age of the sample was 15.5 years old (Table 1). Almost 80% of the sample was White, and 16.6% were Hispanic/Latino. Over three-quarters of the sample were between grades 9 and 11.

Just over 70% of the farm adolescent sample met the criteria for at least mild symptoms of depression (PHQ-A score ≥ 5). There was no significant difference in the distribution of girls and boys by severity of depression symptoms (*p* = 0.155) (Table 2). 

Among anxiety disorders, 62.8% of the farm adolescent sample met the criteria for a probable panic disorder, and 70.5% met the criteria for a probable separation anxiety disorder. There was no significant difference in the prevalence of any anxiety-related disorder between boys and girls (Table 3). 

The mean scores of the stress domains within the Adolescent Stress Questionnaire—Short (ASQ-S) ranged from 1.36 (romantic relationships) to 1.82 (future uncertainty and school performance). Girls reported significantly more stress related to school attendance than boys (*p* = 0.025) (Table 4). 

Farm adolescents that met the criteria for at least mild symptoms of depression (PHQ-A score ≥ 5) reported significantly higher mean stress scores for all ASQ-S domains and a higher mean total ASQ-S score (1.9 vs. 1.0) (Table 5).

Over a quarter (27.7%) of the participants reported carrying a weapon at least 1 day in the past 30 days, and 22.5% reported carrying a weapon on school property at least 1 day in the past month (Table 6). 

Just over 20% of respondents indicated they had been threatened or injured with a weapon at least once in the past 12 months. About half of all respondents reported having been in a physical fight in the past month (Table 7). 

Farm adolescents that met the criteria for at least mild symptoms of depression were significantly more likely to have reported that they carried a weapon (*p* < 0.001), carried a weapon on school property (*p* < 0.001), and avoided going to school because they felt unsafe (*p* < 0.001) in the past 30 days. Farm adolescents that met the criteria for at least mild symptoms of depression were also significantly more likely to have reported being threatened at least one time with a weapon (*p* < 0.001) and having participated in a fight at least one time (*p* < 0.001) in the past 12 months (Table 8). 

Almost a quarter (22.5%) of agricultural youths reported having tried cigarette smoking (Table 9). Girls were slightly younger when they first tried cigarette smoking (9.4 vs. 12.5); however, these differences were not significant. Boys reported smoking more days in the past 30 days than girls, 9.4 vs. 5.7, and more cigarettes on the days they smoked than girls. But again, these differences were not significant. 

About a quarter (22.2%) of agricultural youths reported having had a drink of alcohol other than a few sips (Table 9). There was no significant difference in reported alcohol use, age at first use, number of days alcohol was consumed, or binge drinking participation between boys and girls. 

Agricultural youths who reported at least mild depressive symptoms also reported more overall exposure to smoking “even one puff” (24.4% vs. 18.0%) and reported significantly more days smoking cigarettes and significantly more cigarettes per day in the past 30 days (Table 10). The same relationships with respect to alcohol exposure were weaker and not significant.

## 4. Discussion

The mental health of agricultural producers and workers has been garnering increased attention from Extension, the public health, and healthcare providers. This study describes the mental health, stress, and risk-taking behaviors of a sample of agricultural adolescents in the U.S, a population not well examined despite their participation in agricultural work. 

Almost three-quarters (72.2%) of the adolescents in our sample met the criteria for at least mild symptoms of depression, based on their responses to the PHQ-A. These statistics are considerably higher than the prevalence estimates for the general adolescent population in the U.S. [20,45]. A score of 5 or greater was used to categorize adolescents as having probable depression, and in doing so, the authors acknowledge that the comparisons to the general population are imperfect. In addition, the same instruments are not always used to assess for symptoms of depression among the general population, which creates some challenges in making comparisons. Casting a wide net to identify individuals with even mild symptoms is important, especially when considering the population’s limited access to mental health services. These results underscore the need for family, school, and community interventions that prevent the progression of symptoms, especially amongst individuals with mild symptoms. Over 60% of the respondents met the criteria for a probable panic disorder, and 70.5% met the criteria for a probable separation anxiety disorder. These statistics are also much higher than what has been reported among the general U.S. adolescent population [21,45]. However, this warrants important discussion, especially when considering when the data were collected. The surveys were completed by farm adolescents during the summers of 2021 and 2022, when the world was experiencing the COVID-19 pandemic [46]. Many adolescents had been out of school and learning from home for a year or more, depending on the state and district. Public health guidance emphasized sheltering in place and maintaining small social networks [47]. As such, adolescents may have experienced some anxiety related to going back to school and being separated from family for the first time in over a year.

We did not observe a significant difference in mental health status between farm adolescent boys and girls, but 67% of the respondents were boys, and the lower sample size for girls (N = 96) would have limited the statistical power to some degree. Among the general population, girls typically have worse mental health than boys, including more psychological distress and lower life satisfaction [48]. Girls are better at identifying psychological problems, including anxiety and trauma [49], and are more likely to seek help for their mental health, which might help explain why girls are more likely to be diagnosed for mental health conditions. Help-seeking behaviors should be considered when discussing gender disparities in diagnoses. Furthermore, programs to increase help-seeking among all adolescents could result in early intervention and improved health trajectories into adulthood. The prevalence of symptoms of depression and anxiety in our sample suggests that the mental health of agricultural youths should be considered, and equally prioritized, in discussions about agricultural producers’ stress and mental health.

Stress is a risk factor for mental health conditions among adolescents [23,24,25]. Agricultural youths responded to the Adolescent Stress Questionnaire—Short (ASQ-S), a modified version of the Adolescent Stress Questionnaire. The ASQ-S has been translated, used worldwide, and consistently demonstrates good-to-excellent internal consistency [50,51,52]. Among agricultural youths, school performance, future uncertainty, and school leisure were reported as being the most stressful, whereas teacher interactions and romantic relationships were reported as the least stressful. Observationally, the mean ASQ-S score among farm adolescents was substantially lower than the reported mean score in other youth populations, specifically a UK sample [51]. Similarly, the subscale, or stress domain, scores among farm adolescents were substantially lower than the mean scores of the adolescent samples in the UK [51] and Sweden [50]. These observed differences may be a result of the sampling time. Data from farm adolescents were collected during the summer months (July and August) in 2021 and 2022. This sampling time frame was selected because summer is when adolescents are out of school and more likely to be engaged in agricultural work and experience agricultural exposures. However, the domains related to academics, such as school performance, attendance, and teacher interaction, may not have been relevant to farm adolescents in our sample and resulted in an underestimation of their stress levels. 

Among a sample of Dutch adolescents, girls were more likely to have reported stressors related to interpersonal relationships, such as losing a close friend or a romantic breakup [53]. Similarly, in the U.S., girls have demonstrated more stress during adolescence than boys, specifically around stressors related to interpersonal relationships, including peers, family, and romantic relationships [54,55]. However, in our sample of agricultural youths, significant differences were not observed between the sexes for any stress domain, apart from school attendance. Agricultural girls reported significantly more stress around school attendance. 

Importantly, regardless of sex, the stress domain scores were associated with adverse mental health outcomes in agricultural youths meeting the criteria for at least mild symptoms of anxiety or depression separately, reporting significantly higher stress domain scores. Chronic and acute stress have been identified as predictors of the onset and severity of depression in youths [56]. Among adult agricultural producers, associations between farm-related stressors (time pressures, commodity prices, and weather) and mental health conditions have been observed [9]. Our results provide further evidence that adolescent stress may impact their mental health; however, causality in this relationship cannot be determined based on our study design. Regardless, the relationship between stress and mental health among agricultural youths is similar to what has been observed among agricultural adults. We acknowledge that the ASQ-S does not inquire about agricultural-specific stressors. While there are various instruments to identify or quantify agricultural-related stressors among adults [2,57], these have not been validated among adolescents and are likely not appropriate for an adolescent audience. However, this is an area for future inquiry. An adolescent instrument should consider the various sources of stress for adolescents, including the farm, school, peers, family, and others, such that comparisons across types of stressors can be made. Furthermore, associations between stress and mental health symptoms should be interpreted with caution, given that the recall period was not consistent across instruments. For example, the PHQ-A asks respondents to report on symptoms in the past two weeks, whereas the ASQ-S asks about stressors in the past year [39,50]. While the 12-month recall period might raise concerns about recall bias, traumatic events and stressors are often recalled more easily than more mundane experiences among adolescents. 

Adolescence is often associated with greater risk-taking, and this is considered normal to development. Among adolescents, risk-taking has been linked to clinical and social conditions such as mental health issues, sexually transmitted infection, and violence [58,59]. We observed important and concerning risk-taking behaviors among agricultural youths in addition to associations between mental health and risk-taking. Less than a quarter of our sample reported having tried cigarette smoking or ever having a drink of alcohol (22.5% and 22.2%, respectively). Less than 10% of our sample reported having used an electronic vaping device, ever having used marijuana, or ever having used smokeless tobacco. Among adolescents that participated in the 2021 Youth Risk Behavior Survey, a national survey administered by the CDC, 47.5% reported ever consuming alcohol, and 27.8% reported ever having used marijuana [60]. 

While there have been notable declines in substance use among adolescents, these prevalence estimates are still much higher than what we observed among agricultural youths. Lower reports of substance use among agricultural adolescents may also reflect more limited access for adolescents in rural and agricultural communities. In addition, the lack of anonymity and privacy in small, rural communities with limited retail options may be protective against substance use among agricultural youths. 

The prevalence of violence-related behaviors among agricultural youths in our sample is especially concerning. Just over a quarter of agricultural youths reported carrying a weapon in the past month, and just under a quarter reported carrying a weapon on school property in the past month. Furthermore, about half of the respondents reported having been in a physical fight in the last year. Questions on the farm adolescent survey were pulled from the Youth Risk Behaviors Surveillance Survey (YRBSS), a national public health survey of youths’ risk-taking behaviors, in order to compare our farm sample to the larger, general youth population. In 2021, 3.1% of high school youths surveyed reported having carried a weapon on school property, and 18.3% reported having been in a physical fight in the past year [61]. These results may be more reflective of rural and agricultural youths’ exposure to weapons but not necessarily a propensity towards violence. Rural adolescents are more likely to be exposed to firearms at a young age [62], farm youths are more likely to have some weapons, such as rifles and shotguns, than non-farm youths [63], and they are more likely to participate in recreational sports and activities where weapons are commonplace. 

Among agricultural youths, violence-related behaviors are associated with symptoms of depression. Individuals who met the criteria for at least mild symptoms of depression were more likely to have reported carrying a weapon, carrying a weapon on school property, and avoiding school because of feeling unsafe at least one time in the past 30 days. They were also significantly more likely to have been threatened with a weapon and been in a physical fight at least once in the past year. Given the study design, we cannot establish a causal relationship between these variables. Importantly, expanding too much on these results may perpetuate stigmatizing perceptions that individuals with mental health conditions are dangerous [64]. Research suggests that individuals with mental health conditions are usually targets of violence, which may contribute to the carrying a weapon or participating in a fight among farm adolescents [65]. Regardless of the direction of the relationship, the results related to carrying a weapon, carrying a weapon on school property, and having participated in a fight underscore the need for additional research around how the weapons are being used or the intent. Violence reduction intervention programs, resources, and services should be developed and evaluated in rural and agricultural communities. Recognizing ready access to weapons in rural areas and early exposure [66], programs to emphasize the safe storage of weapons should also be deployed. 

The results of this cross-sectional study of agricultural youths’ stress, mental health, and risk-taking lead to important discussions about the access and availability of resources and services for youth-based resources and services in communities and schools. Most of the agriculture in the U.S. occurs in rural communities that are often medically underserved, especially when it comes to mental health services [67]. Additional barriers to mental healthcare in rural communities include stigma, costs, and time/distance to care, if services are available. Among adolescents, stigma and embarrassment remain pervasive barriers to help-seeking, as well as cost and the availability of resources [68]. Unfortunately, these barriers to help-seeking have also been cited among agricultural adults [69,70]. Parents and/or adult guardians often act as gatekeepers to medical access and care for their adolescent minors [71]. Adults who are distrusting of medical services, perceive stigma, or feel resources are financially inaccessible may instill a similar belief in their children [72] and perpetuate generations hesitant to seek help for mental health concerns.

Furthermore, parents who are experiencing tremendous stress and/or mental health conditions themselves may not be able to recognize signs of mental health conditions in their adolescents. Training other adults who have routine contact with adolescents, such as educators, youth organization leaders, and coaches, could be important to identifying early signs of stress, mental health conditions, and risk-taking behaviors. Programs that increase the awareness of mental health conditions and promote the skills and confidence to intervene should be tested and evaluated among adults in agricultural environments. 

While the results of this study are among the first to describe the sources of stress and mental health experiences of agricultural youths, the results should be interpreted considering some limitations. First, the cross-sectional design of the study limits the results to associations between variables, and causal relationships cannot be assumed. Also, comparisons to non-farm youths must be made and interpreted with caution, as the sample lacks a non-farm control group. Comparisons made are based on the most similar results of the general population. Additional limitations include those consistent with survey research, including recall bias and social desirability bias. The research team’s communication with the participants emphasized confidentiality; however, written consent and assent were required, which may have discouraged some participants from responding truthfully to some survey items. Additionally, survey respondents were recruited via a convenience sample, which may have introduced additional biases. There may be some selection bias in who completed or participated in the study. Parents were required to consent their child to participate, and parents may have been persuaded or dissuaded to participate depending on their own mental health status or personal stigma. Therefore, our sample may not be representative of the broader agricultural population and may over- or underreport the prevalence of mental health conditions among farm youths. Furthermore, we did not inquire about some other potentially important risk factors for mental health conditions among youths, such as gender identity and LGBTQ+ status, which should be included in future studies.

## 5. Conclusions

The mental health of agricultural populations should continue to be a public health priority, with increased attention on youths and families. The agricultural youths in our sample reported worse mental health than published reports for the general population. In addition, a high proportion of agricultural youths participate in risk-taking behaviors related to weapons and violence. Additional research should further examine relationships between risk-taking behaviors and mental health with the goal of understanding causal relationships. Programs and interventions to reduce access to weapons in rural and agricultural communities should also be evaluated. 

## Figures and Tables

**Table 1:** Demographics of farm adolescents by sex (N = 298 ^1^).

Demographic	All N (%) or Mean (SD)	Boys N (%) or Mean (SD)	Girls N (%) or Mean (SD)
Age	15.5 (1.2)	15.5 (1.2)	15.4 (1.3)
Sex	298 (100%)	195 (67%)	96 (33%)
Race			
White	237 (79.5%)	163 (83.6%)	69 (71.9%)
Black or African Am.	47 (15.8%)	26 (13.3%)	21 (21.9%)
Other	15 (5.0%)	6 (3.1%)	7 (7.3%)
Hispanic (Yes)	49 (16.6%)	34 (17.4%)	11 (11.6%)
Education level (current)			
7th	13 (4.4%)	6 (3.1%)	7 (7.3%)
8th	35 (11.7%)	21 (10.8%)	14 (14.6%)
9th	74 (24.8%)	50 (25.6%)	21 (21.9%)
10th	70 (23.5%)	49 (25.1%)	18 (18.8%)
11th	79 (26.5%)	54 (27.7%)	25 (26.0%)
12th	27 (9.1%)	15 (7.7%)	11 (11.5%)
Participate in agricultural work	180 (60.6%)	113 (58.2%)	61 (63.5%)
Mean hours of agricultural work during school year	13.2 (10.9)	13.4 (12.1)	12.9 (8.8)
Mean hours of agricultural work during non-school year	19.6 (13.4)	19.4 (14.3)	19.6 (12.1)
Operator of agricultural operation youth works on			
Parents	168 (93.9%)	107 (94.7)	55 (91.7)
Grandparents	7 (3.9%)	3 (2.7%)	4 (6.7%)
A neighbor, non-relative	1 (0.6%)	1 (0.9%)	0 (0%)
Other relative	3 (1.7%)	2 (1.8%)	1 (1.7%)
In the last 12 months, have you been injured working on the farm or ranch? (Yes only)	21 (11.8%)	17 (15.2%)	3 (5.0%)
Self-rated physical health			
Excellent	104 (35.6%)	70 (36.8%)	34 (35.4%)
Good	165 (56.5%)	105 (55.3%)	54 (56.3%)
Fair	22 (7.5%)	15 (7.9%)	7 (7.3%)
Poor	1 (0.3%)	0 (0%)	1 (1.0%)
Self-rated mental health			
Excellent	82 (27.7%)	58 (29.9%)	24 (25.3%)
Good	164 (55.4%)	109 (56.2%)	48 (50.5%)
Fair	43 (14.5%)	26 (13.4%)	17 (17.9%)
Poor	7 (2.4%)	1 (0.5%)	6 (6.3%)

^1^ A total of 298 returns: Rows may not total 298 due to non-responses.

**Table 2:** Prevalence of symptoms of depression among farm adolescents by sex.

PHQ-A Category and Score	All (N = 298) n (%)	Boys (N = 195) n (%)	Girls (N = 96) n (%)	*p*-Value *
None (0–4)	89 (29.9%)	57 (29.2%)	30 (31.3%)	0.155
Mild (5–9)	93 (31.2%)	67 (34.4%)	23 (24.0%)	
Moderate (10–14)	79 (25.6%)	53 (27.2%)	26 (27.1%)	
Moderately Severe (15–19)	34 (11.4%)	17 (8.7%)	15 (15.6%)	
Severe (20+)	3 (1.0%)	1 (0.5%)	2 (2.1%)	

* Chi-square *p*-values for comparing boys to girls.

**Table 3:** Prevalence of symptoms of anxiety-related disorders among farm adolescents by sex.

Anxiety Related Disorder	All (N = 291)		Boys (N = 195)		Girls (N = 96)		*p*-Value *
	N Meeting Criteria	%	N Meeting Criteria	%	N Meeting Criteria	%	
Panic Disorder or Significant Somatic Symptoms	187	62.8%	120	61.5%	60	62.5%	0.874
Generalized Anxiety Disorder	141	47.3%	85	43.6%	51	53.1%	0.125
Separation Anxiety Disorder	210	70.5%	135	69.2%	69	71.9%	0.643
Social Anxiety Disorder	109	36.6%	71	36.4%	35	36.5%	0.994
School Avoidance	123	41.3%	78	40.0%	39	40.6%	0.919

* Chi-square *p*-values for comparing boys to girls.

**Table 4:** Table of means ^1^, standard deviations, and test score reliability of ASQ-S.

Stress Domain (# Items)	All (N = 291)	Boys (N = 195)	Girls (N = 96)	*p*-Value *	Cronbach’s Alpha
	Mean (SD)	Mean (SD)	Mean (SD)		
Home Life (4)	1.57 (0.86)	1.59 (0.85)	1.51 (0.90)	0.550	0.79
School performance (3)	1.82 (0.86)	1.78 (0.81)	1.88 (0.97)	0.255	0.69
School attendance (2)	1.47 (1.03)	1.35 (0.97)	1.65 (1.11)	0.025	0.71
Romantic relationships (3)	1.36 (0.98)	1.37 (0.93)	1.35 (1.09)	0.752	0.81
Peer pressure (4)	1.64 (0.90)	1.65 (0.88)	1.61 (0.95)	0.620	0.83
Teacher interactions (3)	1.46 (0.88)	1.47 (0.85)	1.42 (0.95)	0.616	0.74
Future uncertainty (3)	1.82 (0.94)	1.75 (0.98)	1.93 (0.97)	0.243	0.82
School leisure (3)	1.71 (0.88)	1.67 (0.88)	1.77 (0.91)	0.403	0.77
Financial pressures (2)	1.66 (1.03)	1.59 (0.97)	1.74 (1.13)	0.219	0.84
Total ASQ-S (27)	1.62 (0.71)	1.59 (0.70)	1.64 (0.74)	0.472	0.95

* Kruskal–Wallis p-values for comparing scores of boys to girls. ^1^ Means and SD are based on averages over domain items on a 0–4-point scale (0 = not at all stressful, 4 = very stressful).

**Table 5:** Association between reported stress by stress domain and depressive symptoms among agricultural youths.

Stress Domain	PHQ-A Score		*p*-Value *
	Score ≤ 4 Mean (SD)	Score ≥ 5 Mean (SD)	
Home life (4)	1.1 (1.0)	1.8 (0.7)	<0.001
School performance (3)	1.3 (1.0)	2.0 (0.7)	<0.001
School attendance (2)	0.8 (1.0)	1.8 (0.9)	<0.001
Romantic relationships (3)	0.7 (0.8)	1.6 (0.9)	<0.001
Peer pressure (4)	1.0 (1.0)	1.9 (0.7)	<0.001
Teacher interactions (3)	0.9 (0.9)	1.7 (0.7)	<0.001
Future uncertainty (3)	1.3 (1.1)	2.1 (0.7)	<0.001
School leisure (3)	1.2 (1.0)	1.9 (0.7)	<0.001
Financial pressures (2)	0.9 (1.1)	2.0 (0.8)	<0.001
Total ASQ-S (27)	1.0 (0.8)	1.9 (0.5)	<0.001

* Kruskal–Wallis tests comparing PHQ-A groups.

**Table 6:** Frequency of weapon related activity among farm youths.

Risk Behavior	0 Days	1 Day	2–3 Days	4 or 5 Days	6 or More Days
	n (%)	n (%)	n (%)	n (%)	n (%)
Carry a weapon such as a gun, knife, or club in the past month?	214 (72.3%)	37 (12.5%)	33 (11.1%)	11 (3.7%)	1 (0.3%)
Carry a weapon such as a gun, knife, or club on school property in the past month?	231 (77.5%)	32 (10.7%)	21 (7.0%)	12 (4.0%)	2 (0.7%)
Not go to school because you felt that you would be unsafe at school or on your way to or from in the past month?	204 (68.7%)	40 (13.5%)	34 (11.4%)	15 (5.1%)	4 (1.3%)
During the past 12 months, on how many days did you carry a gun? (Do not count the days when you carried a gun only for hunting or for a sport, such as target shooting.)	236 (79.5%)	24 (8.1%)	26 (8.8%)	5 (1.7%)	6 (2.0%)

**Table 7:** Threatening and physically violent activities in the past year among agricultural youths.

	0 Times	1 Time	2 or 3 Times	4 or 5 Times	6 or 7 Times	8 or 9 Times	10 or 11 Times	12 or More Times
	n (%)	n (%)	n (%)	n (%)	n (%)	n (%)	n (%)	n (%)
During the past 12 months, how many times has someone threatened or injured you with a weapon such as a gun, knife, or club on school property?	235 (79.4%)	22 (7.4%)	15 (5.1%)	14 (4.7%)	6 (2.0%)	3 (1.0%)	1 (0.3%)	0 (0.0%)
During the past 12 months, how many times were you in a physical fight?	156 (52.3%)	46 (15.4%)	62 (20.8%)	21 (7.0%)	12 (4.0%)	1 (0.3%)	0 (0.0%)	0 (0.0%)

**Table 8:** Association between violent-related behaviors and depressive symptoms among agricultural youths.

Risk-Taking Behavior		PHQ-A Score		*p*-Value *
		Score ≤ 4 n (%)	Score ≥ 5 n (%)	
Carry a weapon such as a gun, knife, or club in the past month?	0 days	82 (93.2%)	132 (63.5%)	<0.001
	At least 1 day	6 (6.8%)	76 (36.5%)	
Carry a weapon such as a gun, knife, or club on school property in the past month?	0 Days	87 (97.8%)	144 (68.9%)	<0.001
	At least 1 day	2 (2.2%)	65 (31.1%)	
Not go to school because you felt that you would be unsafe at school or on your way to or from in the past month?	0 Days	83 (93.3%)	121 (58.2%)	<0.001
	At least 1 day	6 (6.7%)	87 (41.8%)	
During the past 12 months, how many times has someone threatened or injured you with a weapon such as a gun, knife, or club on school property?	0 times	86 (97.7%)	149 (71.6%)	<0.001
	At least 1 time	2 (2.3%)	59 (28.4%)	
During the past 12 months, how many times were you in a physical fight?	0 times	63 (70.8%)	93 (44.5%)	<0.001
	At least 1 time	26 (29.2%)	116 (55.5%)	

* Pearson’s chi-square test of association.

**Table 9:** Reported substance use among agricultural youths by sex.

Substance Use	All n (%) or Mean SD	Boys n (%) or Mean SD	Girls n (%) or Mean SD	*p*-Value *
Have you ever tried cigarette smoking, even one puff?—Yes only	67 (22.5%)	46 (23.6%)	19 (19.8%)	0.550
How old were you when you first tried cigarette smoking, even one or two puffs?	13.1 (2.2)	13.4 (2.0)	12.5 (2.6)	0.132
During the past 30 days, on how many days did you smoke cigarettes?	8.1 (8.0)	9.4 (8.8)	5.7 (5.1)	0.153
During the past 30 days, on the days you smoked, how many cigarettes did you smoke per day?	3.5 (4.0)	4.0 (4.4)	2.6 (3.0)	0.185
Have you ever used an electronic vapor product?—Yes only	25 (8.4%)	18 (9.2%)	7 (7.3%)	0.661
How old were you when you first tried an electronic vapor product?	14.0 (1.8)	13.7 (1.8)	14.6 (1.6)	0.277
During the past 30 days, on how many days did you use an electronic vape?	4.9 (5.6)	6.2 (6.1)	1.7 (1.6)	0.078
How old were you when you first tried a smokeless tobacco product?	13.7 (1.8)	13.3 (1.3)	14.3 (2.5)	0.589
During the past 30 days, on how many days did you use a smokeless tobacco product?	1.5 (1.9)	2.0 (2.2)	0.5 (0.7)	0.340
Have you ever used marijuana?—Yes only	20 (6.7%)	13 (6.7%)	7 (7.3%)	1.000
How old were you when you first tried marijuana for the first time?	14.3 (2.0)	14.5 (1.9)	13.9 (2.3)	0.628
During the past 30 days, on how many days did you use marijuana?	7.5 (9.4)	4.8 (7.0)	12.1 (11.6)	0.287
Have you ever had a drink of alcohol other than a few sips?—Yes only	66 (22.2%)	41 (21.1%)	21 (21.9%)	1.000
How old were you when you had your first drink of alcohol other than a few sips	13.1 (2.1)	13.4 (2.3)	12.7 (1.9)	0.151
During the past 30 days, on how many days did you have at least one drink of alcohol?	4.7 (4.9)	4.4 (3.8)	5.8 (6.7)	0.841
During the past 30 days, on how many days did you have 4 more drinks of alcohol (girls)/have 5 or more drinks of alcohol (boys) within a couple of hours?	1.5 (2.0)	1.3 (1.6)	2.1 (2.5)	0.170

* Pearson’s chi-square test of association (questions summarized with %) or Kruskal–Wallis tests comparing boys to girls.

**Table 10:** Association between substance use and depressive symptoms among agricultural youths.

Substance Use		PHQ-A Score		*p*-Value
		Score ≤ 4 n (%) or Mean (SD)	Score ≥ 5 n (%) or Mean (SD)	
Have you ever tried cigarette smoking, even one puff?	Yes	16 (18.0%)	51 (24.4%)	0.231 *
	No	73 (82.0%)	158 (75.6%)	
During the past 30 days, on how many days did you smoke cigarettes?		4.5 (7.6)	9.3 (7.9)	0.006 ^#^
During the past 30 days, on the days you smoked, how many cigarettes did you smoke per day?		1.4 (1.9)	4.2 (4.3)	0.002 ^#^
Have you ever had a drink of alcohol other than a few sips?	Yes	17 (19.1%)	49 (23.6%)	0.448 *
	No	72 (80.9%)	159 (76.4%)	
During the past 30 days, on how many days did you have at least one drink of alcohol?		3.4 (2.7)	5.2 (5.4)	0.353 ^#^
During the past 30 days, on how many days did you have 4 or more drinks of alcohol (girls)/have 5 or more drinks of alcohol (boys) within a couple of hours?		0.9 (1.1)	1.8 (2.2)	0.170 ^#^

* Pearson’s chi-square test of association. ^#^ Kruskal–Wallis tests comparing PHQ-A groups.

## Data Availability

Data are not currently available.
